# Supplementary material for: Lipoprotein Particles as Shuttles for Hydrophilic Cargo
Source: Membranes (Basel). 2023 Apr 28;13(5):471. doi: 10.3390/membranes13050471 (PMC10222575; doi:10.3390/membranes13050471)
Supplement: Supplementary file 1 [file membranes-13-00471-s001.zip › membranes-2334361-supplementary.pdf]

Supplement Information

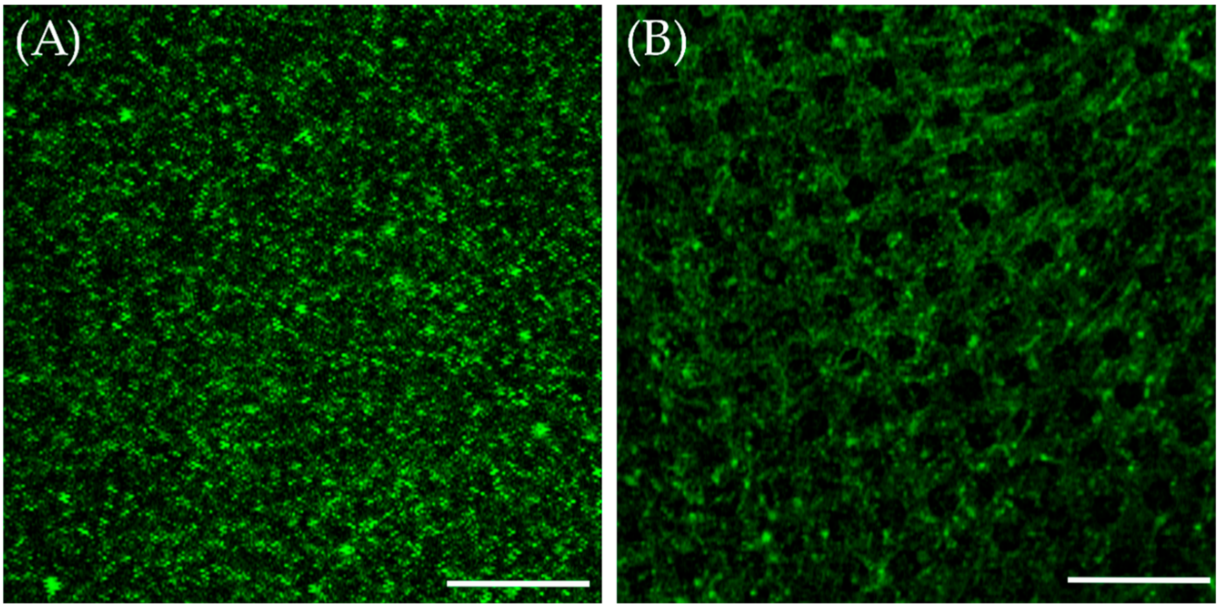

Figure S1: Functionalized HDL surfaces. (A) Homogeneous immobilization of HDL loaded particles and (B) micro-contact printed loaded HDL particles. Scale bar = 20 µm

Two approaches were chosen to control the density of the HDL particles to be immobilized. Thus, the glass surface was coated with APTES. In the first case, HDL particles loaded with 18:1 DGS NTA (Ni) lipid (5 mol%) were incubated directly onto the test surface, in the second case these particles were positioned via micro-contact printing [1][2], in each case with a concentration of 100 µg/mL. Briefly, micro-contact printing generates areas enriched or depleted in the protein of interest. Commonly, this method is used to identify and quantify the interaction of a membrane protein ("bait") and a fluorescently labeled interaction partner ("prey") in live cells. Therefore, PDMS stamps with a three-dimensional defined structure, are inked with protein solution, washed, and transferred onto glass slides. Wherever the stamp touches the surface, the proteins are bound to the surface; places where there is no contact are free of proteins. This was intended to ensure that the interaction surface with HDL particles was reduced by half for subsequent exchange studies and hence a decrease in the transferable lipids as well. Both surfaces resulted in saturation of maximally transferred lipid-anchored insulin molecules from the HDL particle into the contacting GUV membrane. Consequently, the same amount of DGS NTA(Ni)-His$_6$-insulin changes the target membrane within 10 minutes, regardless of the insulin density offered. Both surfaces showed saturation. After immobilization, insulin-His$_6$ was added to attach the particle and then unbound ones were removed by multiple washing steps. To visualize the insulin molecules microscopically, an antibody against insulin together with a fluorescent label was used and purified in the analogous step as before.

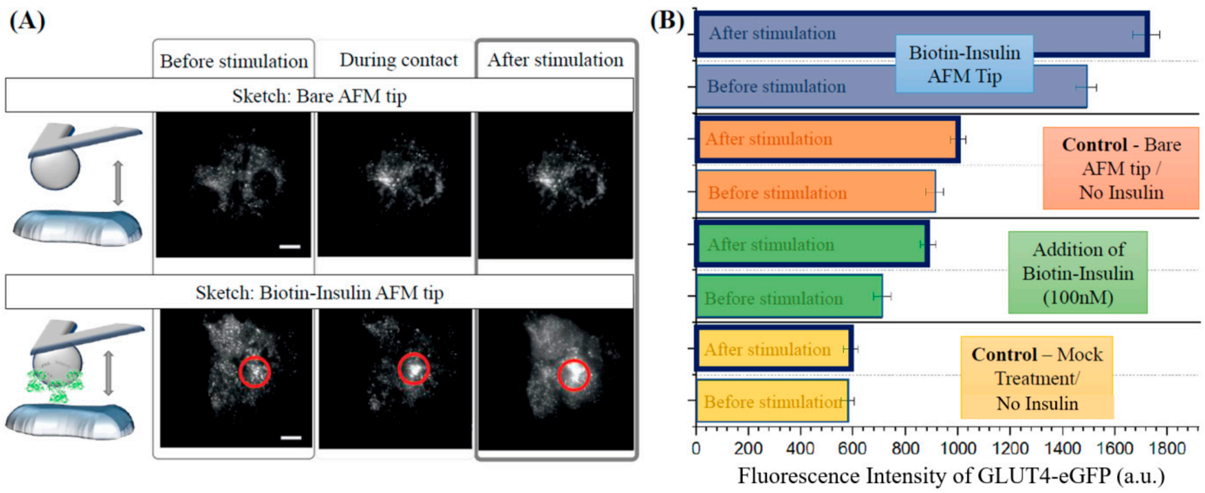

Figure S2. (A) Punctual stimulation via a bare spherical AFM tip (negative control-upper sketch) and a spherical biotin-insulin tip (bottom sketch). Representative fluorescence images of the Glut4-eGFP signal, before, during and after stimulation via the respective AFM tips. The red circle depicts the position of the spherical functionalized AFM tip. Intensity is equally scaled. Time between attachment and detachment is 20 minutes. Scalebar: 10 µm. (B) Mean fluorescence intensity of the entire cell (averaged per pixel) of translocated GLUT4-eGFP (counts in arbitrary units) before and after treatment. Each bar indicates three cells. Yellow: cells were mock treated; green: Addition of biotin insulin (100 nM) to the cells for 20 minutes; orange: Stimulation via bare AFM tip; blue: Stimulation via biotin-insulin tip for 20 minutes.

<u>Punctual cell stimulation – experimental detail to Figure S2:</u>

AFM probes were incubated with 30 µL (1 mg/mL) streptavidin and subsequently washed by rinsing with PBS and afterwards incubated with biotin-insulin at a concentration of 100 µg/mL for 30 minutes. After incubation the cantilever was washed by rinsing with PBS. After starving, the cells were placed on the combined SMFM/AFM microscope and acclimatized for 30 minutes at room temperature. With the aid of the combined technique, the insulin tip is contacted with defined forces on the cell membrane in a controlled manner and at the same time the response of the translocation was monitored via the fluorescence signal. In particular, the insulin coated tip was directed to the cell membrane for punctual stimulation. Thus, the insulin tip is offered to the cell at precisely defined locations on the membrane and the translocation of the receptor was visualized using fluorescence microscopy of GLUT4-eGFP molecules (Figure S2 – left). The functionalized AFM tip was brought in contact for 20 minutes and was afterwards removed. The fluorescence signal was measured before, during and after contact (Figure S2 – right). A negative control without using insulin was performed by using a bare tip. To extract the percentual increase of GLUT4-eGFP on the cell membrane via punctual stimulation compared to whole cell-surface stimulation, additionally the cell was directly incubated with a 100 nM biotin-insulin solution for 20 minutes. Again, the intensity of translocated GLUT4-eGFP was measured before and after stimulation. For control we followed the identical procedure by mock treating the cells. CHO-K1-hIR/GLUT4-myc-GFP cells showed no translocation of the receptor in the control treatment, however by addition of biotin-insulin (final concentration 100 nM), the signal of the receptor increased by about 20%. A similar increase in the translocation of GLUT4-eGFP was observed by local stimulation via biotin-insulin tip. A negligible increase in the signal was observed when stimulating with the naked tip. Not unexpectedly, the measurements with the AFM tip showed an increase in autofluorescence, which caused a general increase in background.
